# Comprehensive secondary analysis of thrombotic events in pediatric patients receiving extracorporeal membrane oxygenation: A prospective cohort study

**DOI:** 10.1177/02676591241289358

**Published:** 2024-10-01

**Authors:** Tyler B Hughes, Rebecca N Treffalls, Wouter Koek, Heidi Dalton, Oliver Karam, Andrew D Meyer

**Affiliations:** 1 Division of Critical Care, Department of Pediatrics, Long School of Medicine, 12345 University of Texas Health Science Center, San Antonio, TX, USA; 2 School of Medicine, 7079 University of Incarnate Word, San Antonio, TX, USA; 3 Adult and Pediatric ECMO, 3313 INOVA Fairfax Medical Center, Fairfax, VA, USA; 4 Department of Pediatrics, 12228 Yale School of Medicine, New Haven, CT, USA

**Keywords:** extracorporeal membrane oxygenation, thrombosis, pediatric, transfusion, mortality, thrombosis risk

## Abstract

**Introduction:**

This study aims to describe laboratory and clinical factors associated with thrombotic events during prolonged pediatric extracorporeal membrane oxygenation.

**Methods:**

A secondary analysis of a multi-center prospective study performed between 2012 and 2014. Patients under the age of 19 years that received extracorporeal membrane oxygenation for at least 4 days of therapy were included (*n* = 385). Univariable analysis and binomial regression were performed to evaluate predictive factors of single and multiple thrombotic events. A posteriori scoring tool was created to categorize thrombotic event severity.

**Results:**

Over 39% of children receiving prolonged ECMO experienced a thrombotic event (TE). Binomial regression demonstrated an association between higher transfused platelet volume (mL/kg) (OR 1.04, CI: 95% 1.01–1.06, *p* = 0.003), Anti-Xa (OR 5.38, CI: 95% 1.22–23.8, *p* = 0.026) and aPTT (OR 1.01, CI: 95% 1.00–1.02, *p* = 0.032) the day prior to TE. Patients experiencing multiple TEs were associated with higher platelet transfusion volume (mL/kg) (OR 1.08, CI: 95% 1.05–1.12, p =< 0.001), antithrombin III (OR 1.03, CI: 95% 1.01–1.04, *p* = 0.001) and aPTT (OR 1.02, CI: 95% 1.01–1.03, *p* = 0.009). Patients experiencing multiple thrombotic events had a higher risk of 28-day mortality based on a cumulative clot severity score >4 (OR 2.37 (CI: 95% 1.32–4.24).

**Conclusions:**

Current lab tests show limited sensitivity to predict these events the day prior in a vulnerable patient group, leading to potential ECMO circuit failures. Patients with multiple thrombotic events during ECMO therapy face increased mortality risks, highlighting the need for dynamic reporting tools like clot severity scores and detailed documentation of interventions to enhance understanding and improve outcomes.

## Introduction

Pediatric extracorporeal therapy can provide lifesaving therapy for patients with organ failure recalcitrant to conventional management.^1^ Extracorporeal membrane oxygenation (ECMO) provides cardiac and/or respiratory life support using an artificial lung (oxygenator), mechanical blood pump, and arterial and venous cannulas. Unfortunately, multiple factors including foreign material of the ECMO system, underlying disease pathology, and alteration of the patient’s innate hemostasis result in thrombosis.^2^ Attempts to mitigate circuit thrombosis includes systemic anticoagulation and use of coated materials.^3^ Balancing risk of thrombosis versus bleeding from systemic anticoagulation remains one of the most challenging aspects for patients receiving ECMO therapy. Despite an increase in utilization from the COVID-19 epidemic, incorporation of direct thrombin inhibitors and anticoagulation research, heparin remains the primary mode of anticoagulation and complications from coagulopathy persist.^4–6^ Over 38% of children supported on ECMO will have a thrombosis effecting the patient or the circuit.^4,7^

Investigating risk factors associated with thrombosis include evaluation of ECMO circuit parameters, systemic anticoagulation lab values and required blood product administration.^6,8–10^ Escalating ECMO circuit parameters such as pump pressure, pressure differential across an oxygenator, blood flow to maintain cardiac output, or oxygen support without clinical change in the patient may be an early sign of circuit thrombosis.^10^ The predictive values of standard anticoagulation markers, such as international normalized ratio (INR), activated partial thromboplastin clotting time (aPTT), activated clotting time (ACT), anti-factor Xa (anti-Xa), plasma-free hemoglobin (PFH), D-Dimer, have only been evaluated in small trials.^7,8,11,12^ These laboratory tests are correlated with visual inspection of the ECMO circuit to assess for thrombosis location and need for intervention.^3,13^ However, these are reactionary strategies to evolving thrombotic events.^4,8,10^ Thrombosis can result in local ischemia or embolism to a patient’s vital organs. Thrombosis to the circuit or circuit components can decrease efficacy of ECMO therapy resulting in inadequate support.^2,3^ Studies evaluating risk of thrombotic events comprehensively remain scarce with many focused on bleeding and associated complications.^6,9,14^ Although bleeding is a significant cause of mortality, bleeding risk reduction by decreasing anticoagulation comes at the cost of increased risk of thrombosis and associated complications. Despite ECMO being so frequently used, there is currently a paucity of studies evaluating risk of thrombotic events in a comprehensive way. To set a foundation for further studies, we performed a secondary analysis on a large pediatric ECMO population database to assess the predictive utility of current laboratory testing for single and multiple thrombotic events. As a secondary measure we also evaluated how clot severity may contribute to clinical outcomes in pediatric patients.

## Methods

The Institutional Review Board of UT Health San Antonio approved this study as non-human research using deidentified datasets via data utilization agreement with University of Utah on behalf of Collaborative Pediatric Critical Care Research Network (CPCCRN); Protocol HSC20190118N, 19 February, 2019. The BATE database was a prospective observational study involving eight institutions of the CPCCRN between December 2012 to September 2014.^4^ Centers enrolled consecutive patients under 19 years of age receiving ECMO treatment. Patients from pediatric, neonatal, or pediatric cardiac intensive care units (PICU, NICU, CICU) were included. Patients receiving several non-continuous runs of ECMO therapy had only the first therapy duration included in the data. The BATE database is composed of 514 enrolled ECMO pediatric patients. The primary objectives of the BATE study were to measure the incidence of bleeding and thrombosis during ECMO support, identify factors associated with these complications and determine the impact of these complications on patient outcome.

**Figure 1.**
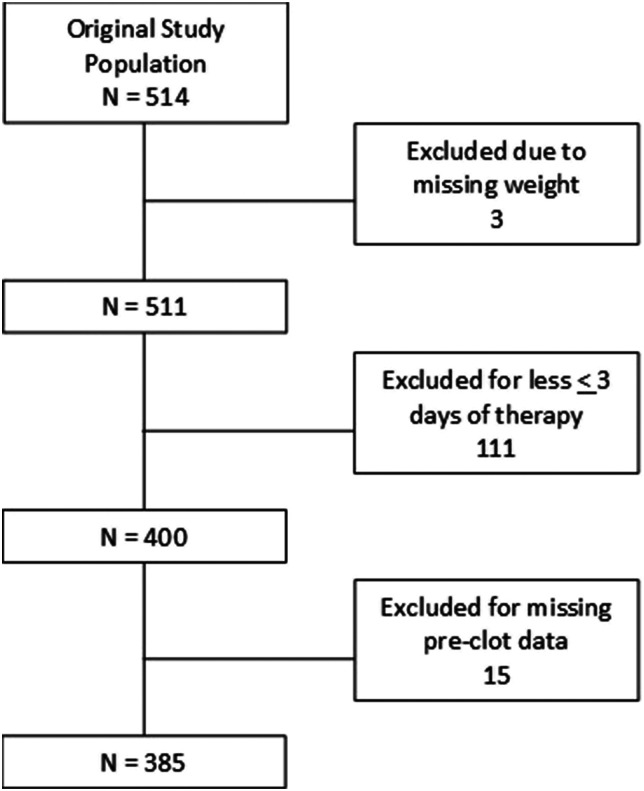
Secondary analysis population.

Inclusion criteria for our analysis included completed demographics including weight, and a prolonged ECMO course (greater than 3 days). To perform pre-thrombotic analysis, patients were also required to have laboratory data from the day prior to their first thrombotic event. The included patient population assessed consisted of 385 patients between the ages 0 days to 19 years (Figure 1).

Demographic and pre-ECMO characteristic data were collected including sex, age, weight, history of prematurity, primary diagnosis, chronic diagnosis, and baseline labs. The original database analysis categorized the population between^1^ neonate (0 to 30 days) and^2^ pediatric (greater than 30 days). To better assess physiologic differences and compare patients more appropriately, groups were further divided to (1) neonate (0 to 30 days of life), (2) infant (30 days to 24 months), (3) child (2 years to 12 years), and (4) adolescents (greater than 13 years) using the Pediatric Risk of Mortality (PRISM) III scoring age groups.^4,15^ Prematurity was defined as less than 37 weeks gestation at birth and reported for the neonate population only.

ECMO data included primary indication (respiratory, cardiac, extracorporeal cardiopulmonary resuscitation [ECPR]), location of deployment (PICU, NICU, CICU), mode of ECMO (venovenous (VV) or venoarterial (VA)), cannula sizes (drain and return), location of cannula (drain and return), and circuit flow at time of laboratory collection. Laboratory data was obtained daily for each patient, including complete blood count, INR, aPTT, ACT, antithrombin III (ATIII), anti-Xa, PFH and fibrinogen. Daily collected data also included amount of transfusion adjusted for weight including average packed red blood cells (pRBC) per kg, plasma per kg, platelet per kg, and total transfusion per kg. Gastrointestinal and chest tube bleeding were also assessed in context of thrombosis. Circuit thrombosis requiring a component change, as well as patient thrombotic events including intracranial infarct, limb ischemia, pulmonary embolus, intracardiac thrombus, aortopulmonary shunt thrombus, or other sites of thrombosis not specified were recorded for the first 28 days. Laboratory data, circuit flow, and daily interventions were separated by subject and categorized into thrombotic event (TE) versus no thrombotic event (NTE). The data from the NTE group were averaged over their ECMO course to create a control group for comparison to the TE group. The data from the TE group were separated into day before clot, and day of clot to perform a clot formation prediction analysis using laboratory and clinical data.

Due to limited descriptive reported data, established thrombosis scoring tools could not be used.^16^ An a posteriori value for severity of thrombotic event was set prior to analysis. Clot severity assigned from 0, no thrombotic event, 1 (mild) involving peripheral components requiring intervention, 2 (moderate) involving more proximal and required components, 3 (severe) a patient experiencing a thrombotic event. For example, a patient experiencing one oxygenator exchange and tubing change within 28 days would receive a score of 3. Scoring was cumulative throughout patient course of ECMO therapy.

### Statistical analysis

GraphPad Prism Version 8.0.0 (GraphPad Software, San Diego, CA, USA) and *jamovi* Version 2.2 (*jamovi* Project, Sydney, NSW, Australia) were used for analysis. Descriptive analysis of demographic and ECMO characteristics were performed. Pearson Chi-squared or Fisher exact test and Student *t* test or Wilcoxon-Mann-Whitney tests were used for univariate analysis. Binomial logistic regressions were performed to determine a model of risk prediction for (1) at least one thrombotic event <28 days and (2) multiple thrombotic events <28 days. The null odds ratio (OR) was set a priori as a value of 1 and statistical significance was defined as *p <* 0.05. The cut-off value for the area under the curve (AUC) was 0.5. Data are presented as mean and confidence interval of the mean 95% (CI 95%). 28-day mortality was assessed with chi-squared test with a clot severity score cutoff of >4. The cutoff was selected to assess both single versus multiple thrombotic events and weight the severity of thrombotic events. Survival curve analysis was performed using log-rank test and log-rank test for trend.

## Results

The BATE database enrolled a total of 514 patients, with 385 patients meeting our criteria for analysis (Figure 1). 111 patients received less than 3 days of ECMO with 31 (28%) deceased and 16 experiencing a thrombotic event (7%). 61 patients were removed from ECMO by day 2 of therapy limiting population size for comparison within the first 3 days. To assess pre-thrombotic event data, 15 patients who experienced a thrombotic event on day 1 were also excluded as no pre-thrombotic data was available for these patients. The median age of the overall cohort was 5 days old, with a median weight of 3.66 kg. Neonates were the most common age group (55.3%) followed by infants (23.6%), children (11.6%), and adolescents (9.4%). Most patients were male (57.9%), Caucasian (46.2%) with 64.9% of patients identified as Non-Hispanic or Latino ethnicity. A large portion of the population was either unknown or not reported by race (29.4%) or ethnicity (17.9%). The primary diagnoses were composed of respiratory distress or failure (49.4%) and congenital cardiovascular disease (31.7%). The median duration of ECMO therapy was 8 days. The study population comprised 82.0% venoarterial and 18.0% venovenous ECMO support (*p =* 0.030). The most frequent indications for ECMO were respiratory failure (50.6%) followed by cardiac failure (38.4%) and emergent cardiopulmonary resuscitation (ECPR) (10.9%). There were 170 patients (44.1%) placed on ECMO in the cardiac intensive care unit (CICU). The most common ECMO circuit component to be changed was the whole circuit (N = 50), followed by other (N = 36), and oxygenator (N = 24). A summary of procedural characteristics is provided in Table 1, full demographic data in supplement.

**Table 1.** Procedural characteristics and outcome data of pediatric patients on ECMO therapy.

Variable	Thrombotic event		*p* value
	Yes (*N* = 155)	No (*N* = 230)	
Mode of ECMO, *n* (%)			0.031
Venovenous	20 (12.9)	50 (21.7)	
Venoarterial	135 (87.1)	180 (78.3)	
Primary ECMO indication, *n* (%)			0.859
Respiratory	81 (52.3)	114 (49.6)	
Cardiac	57 (36.7)	91 (39.6)	
E-CPR	17 (11.0)	25 (10.8)	
Conversion from CPB to ECMO, *n* (%) (VA only)			0.334
Yes	23 (17.0)	23 (12.8)	
No	112 (83.0)	157 (87.2)	
Location of ECMO, *n* (%)			0.686
NICU	55 (35.5)	75 (32.6)	
PICU	31 (20.0)	54 (23.5)	
CICU	69 (44.5)	101 (43.9)	
ECMO return site, *n* (%)			0.002
Carotid artery	89 (57.4)	121 (52.6)	
Femoral artery	1 (0.60)	8 (3.50)	
Great vessels	39 (25.2)	51 (22.2)	
Jugular vein	20 (12.9)	51 (21.7)	
Other	6 (3.90)	0 (0.00)	
ECMO flow rate (mL/min) median, IQR	438 (329–720)	442 (319–975)	0.545
Mortality, *n* (%)	77 (49.7)	84 (36.5)	0.012

ECMO: extracorporeal membrane oxygenation; E-CPR: extracorporeal cardiopulmonary resuscitation; CPB: cardiopulmonary bypass; NICU: neonatal intensive care unit; PICU: pediatric intensive care unit; CICU: cardiac intensive care unit; great vessels include, aorta, pulmonary artery, subclavian and innominate; IQR: interquartile range.

### Univariate analysis

TE patients were more likely to be on VA ECMO compared to NTE (VA, 43.0 % vs 56.0 %; VV 27.0 % vs 73.0 %; *p* = 0.02). Additionally, patients with carotid artery ECMO return sites were higher in the patients with thrombotic events (58.0% vs 54.0%, *p* = 0.35). There were 14.6% of patients that were converted from cardiopulmonary bypass to ECMO that had no significant differences in thrombotic events (17.1% vs 12.7%; *p* = 0.33). The mortality rate in the overall cohort was 42.0% with a significantly higher mortality rate in patients with thrombotic events (49% vs 37%, *p* = 0.02). There were no statistical differences in sex, race, ethnicity, primary diagnosis, ECMO indication, or location of ECMO. Patients with thrombotic events had a higher daily anti-Xa level (0.38 vs 0.28; *p* = 0.003) and higher daily documented PFH (51.6 vs 43.3; *p* = 0.026) compared to those with no thrombotic events. TE patients also reported a higher ATIII activity (65.0 vs 59.0; *p* = 0.031). Patients with thrombotic events received higher amounts of platelets (mL) transfused per kg (16.9 vs 11.6; *p*= <0.001), red blood cell (mL) transfused per kg (17.6 vs 13.6; *p* = 0.001), plasma (mL) transfused per kg (7.38 vs 4.51; *p* = <0.001) and cryoprecipitate (mL) transfused per kg (4.00 vs 0.00; *p* = <0.001). Patients with thrombotic events received a higher daily heparin dosing (units) per kg per day (654 vs 571; *p* =< 0.001) than those that did not experience a thrombotic event. There was no statistically significant difference for daily reported result between the cohorts for circuit flow (mL/min), hours of heparin held the day prior, hemoglobin, hematocrit, platelet count, white blood cell count, PT, INR, aPTT, fibrinogen, or ACT.

### Multivariable analysis

Binomial regression using demographics and documented lab values from the day prior to a thrombotic event, patients in the TE group received more platelet volume (mL/kg) (OR 1.04, CI: 95% 1.02–1.06, *p* = 0.003), higher aPTT (OR 1.01, CI: 95% 1.00–1.92, *p* = 0.032) and noted a higher anti-Xa (OR 5.38, CI: 95% 1.22–23.8, *p* = 0.026) the day prior to their first thrombotic event compared to the NTE group (Table 2). An area under the curve (AUC) of 0.689, specificity 0.887 and sensitivity 0.283 was predicted with a cutoff value set at 0.5. Other demographics such indication for ECMO, cannula size, weight, chest tube or gastrointestinal bleeding, other blood products or coagulation medications (ATIII, Novo7, Amicar) were not statistically significant.

**Table 2.** Binomial regression of first thrombotic event in pediatric patients on ECMO therapy.

Variable	Thrombotic event		
	OR	95% CI	*p* value
Age			
Neonate	Ref		Ref
Premature neonate	2.34	0.71–7.76	0.165
Infant	1.41	0.67–2.97	0.361
Child	1.31	0.59–2.90	0.500
Adolescent	0.83	0.29–2.39	0.735
Ethnicity			
Not hispanic or latino	Ref		Ref
Hispanic or latino	0.49	0.22–1.08	0.076
Unknown not reported	1.29	0.61–2.75	0.51
Sex			
Male	Ref		
Female	1.02	0.57–1.82	0.939
Mode of ECMO			
Venovenous	Ref		Ref
Venoarterial	1.42	0.61–3.32	0.412
Laboratory and transfusion values			
aPTT (s)	1.01	1.00–1.02	0.032
Anti-xa (units/mL)	5.38	1.22–23.8	0.026
Platelet/kg (ml/kg)	1.04	1.01–1.06	0.003

OR: odds ratio; CI: confidence interval; Ref: reference; ECMO: extracorporeal membrane oxygenation.

Assessing for demographics and daily lab values for patients at risk of multiple thrombotic events (MTE) during their ECMO therapy. Seventy-eight patients experienced multiple thrombotic events. Age group and mode of ECMO were not independently associated with increased risk of multiple thrombotic events. MTE patients were noted to have a higher anti-Xa (0.41 vs 0.28; *p* = 0.002), higher ATIII (71.1 vs 59.0; *p* = 0.011) and higher PFH (55.7 vs 43.3; *p* = 0.011). These patients also received more blood products per kilogram: platelets (mL/kg, 19.5 vs 11.6; *p* =< 0.001), red blood cells (mL/kg, 19.6 vs 13.6; *p* =< 0.001), plasma (mL/kg, 9.42 vs 4.51; *p* =< 0.001), cryoprecipitate (mL/kg, 4.89 vs 0.00; *p* =< 0.001), and daily heparin dose (677 vs 571; *p* =< 0.001). Other laboratory values were not independently associated with MTE (Table 3).

**Table 3.** Binomial regression analysis of multiple thrombotic events in pediatric patients on ECMO therapy.

Variable	Multiple thrombotic events		
	OR	95% CI	*p* value
Age			
Neonate	Ref		Ref
Premature neonate	1.37	0.34–5.59	0.663
Infant	1.47	0.57–3.81	0.427
Child	0.97	0.30–3.15	0.961
Adolescent	0.83	0.13–5.15	0.845
Ethnicity			
Not hispanic or latino	Ref		Ref
Hispanic or latino	0.88	0.33–2.39	0.803
Unknown not reported	1.96	0.69–5.69	0.202
Sex			
Male	Ref		Ref
Female	1.10	0.51–2.39	0.804
Mode of ECMO			
Venovenous	Ref		Ref
Venoarterial	1.0.7	0.40–2.88	0.887
Laboratory and transfusion values			
aPTT (s)	1.02	1.01–1.03	0.009
ATIII (%)	1.03	1.01–1.04	0.001
Platelet/kg (mL/kg)	1.08	1.05–1.12	<0.001

OR: odds ratio; CI: confidence interval; Ref: reference; ECMO: extracorporeal membrane oxygenation.

A binomial regression of the MTE population demonstrated an elevated aPTT (OR 1.02, CI: 95% 1.01–1.03, *p* = 0.009), elevated ATIII (OR 1.03, CI: 95% 1.01–1.04, *p* = 0.001) and higher volume of platelet transfusion per kg (OR 1.08, CI: 95% 1.05–1.12 , *p* < 0.001). An area under the curve (AUC) for the receiver operator curve of our model was 0.785 with specificity of 0.915 and sensitivity of 0.404 was predicted.

**Figure 2.**
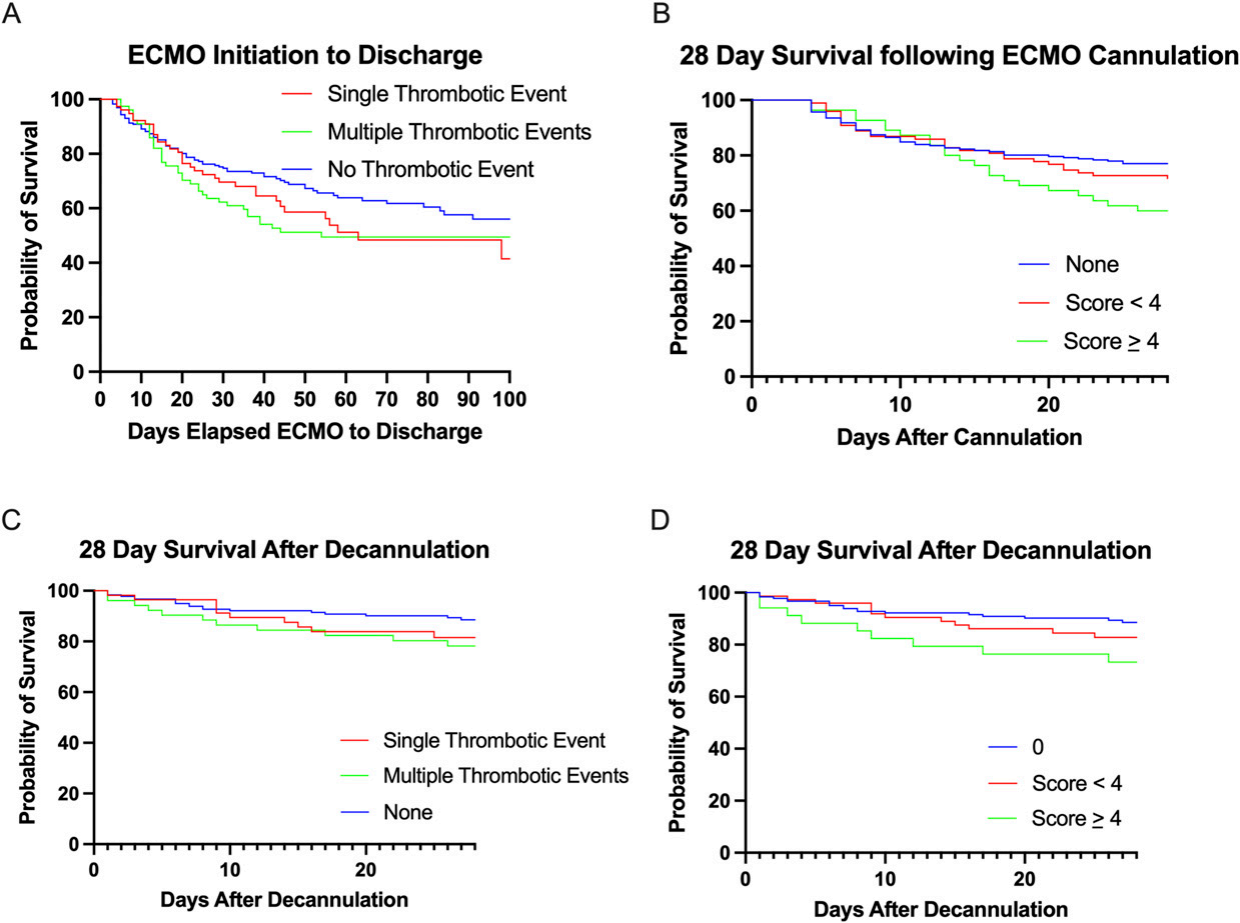
(A) Survival curve of elapsed time in days from ECMO cannulation for patients who experienced no thrombotic events, single thrombotic events, or multiple thrombotic events. (B) 28-days survival after ECMO cannulation based on clot severity score. (C) 28-days survival after decannulation from ECMO based on thrombotic events experienced during ECMO therapy. (D) 28-days survival after decannulation from ECMO based on clot severity score during ECMO therapy.

The maximum number of adverse patient TEs experienced by a patient were two. The maximum number of circuit TEs experienced by a patient were four. Patients with a clot severity score less than 4 experienced a hospital mortality 38.8 %, while patients with a clot severity score greater than or equal to 4 experienced a hospital mortality of 60.0%. Analysis using a contingency table demonstrated cumulative score >4 was associated with increased hospital mortality OR 2.37 (CI 95% 1.32–4.24). Survival analysis was performed using log-rank test (TE vs NTE) and log-rank test for trend (multiple groups: TE, NTE, and NTE; clot scoring clusters). Log-rank testing did not demonstrate statistical significance for mortality over 100 days between patients experiencing no thrombotic event, at least one thrombotic event (*p* = 0.059) or for 28 days mortality following decannulation (*p* = 0.079). Accounting for clot severity, log-rank test for trend was significant for 28-days mortality from cannulation and 28-days mortality after decannulation (*p* = 0.031 and *p* = 0.018). See Figure 2 for survival curves.

## Discussion

Our analysis provided a comprehensive evaluation of underlying patient demographics including cannulation sites, cannula size, flow rates, and types of thrombosis. We specifically investigated a patient population with prolonged ECMO course demonstrating no increased thrombotic events according to age, gender, ethnicity, race, primary diagnosis, primary indication for ECMO, location of cannulation, or flow rate. Daily standard laboratory values were not statistically significant to alert for impending thrombosis. Elevations in ATIII, anti-Xa, and PFH though statistically significant do not elicit strong clinical significance historically. Assessing for daily standard laboratory value differences in patients who experienced multiple thrombotic events during ECMO therapy, an elevated aPTT, ATIII and platelet transfusion volume per kg were noted. Binomial regression did demonstrate an elevated aPTT, anti-Xa and transfused platelet volume per kg the day prior to thrombotic events. Other blood product transfusions, chest tube and/or gastrointestinal bleeding, and incorporation of univariable significant lab values did not improve our regression model. Due to static nature of the database, intervention by the care team to prevent thrombosis as it develops in the circuit and/or patient by increasing anticoagulation (increased anti-Xa, aPTT) the day prior can only be theorized. These regression models though specific were not sensitive enough to predict thrombotic events.

A previous secondary assessment of the database reported that platelet transfusions the day prior were associated with increased thrombotic events.^9^ We expanded upon this assessment comparing patients who experienced multiple thrombotic events during their ECMO course. Previous studies have also listed bleeding as a major predictor of mortality in pediatric patient’s on ECMO.^14^ Scoring clot severity with a non-validated scoring tool was associated with an increased mortality in patients experiencing multiple thrombotic events within 28-days of ECMO cannulation and 28 days after decannulation. As far as we know this is the first attempt to stratify clot severity and its relationship with hospital mortality in patients experiencing prolonged ECMO therapy. Incorporating a scoring tool for clot severity could lead to more specific associations within the ECMO therapy patient population and eventually more targeted interventions.

Several limitations must be acknowledged in evaluating this database and the challenges of translating these findings to real-world application. Transfusion goals were reported in this database but not analyzed in part due to not being consistently updated daily. Clot severity scoring system like the CTCAE would be an ideal scoring system to implement with data collection due to its dynamic nature of thrombosis with and without intervention.^16^ However, as the database only documented the patient or circuit location of thrombosis and day of intervention performed the CTCAE’s utility is limited. The age of this database must also be accounted for as well. Historically ATIII was administered on the first day of ECMO therapy for certain pediatric populations.^17,18^ 64 patients received ATIII on the first day of therapy, it was not found to be associated with increased thrombotic events though practice now differs today. Institutional anticoagulation practices regarding therapeutic agents (Heparin, Bivalirudin, Argatroban) and monitoring continue to differ worldwide, and incorporation of direct thrombin inhibitors must be acknowledged. Heparin was the only anticoagulation therapy used at these centers. However, heparin use continues for the majority institutions as recently as 2020.^5,6,19^ The limitations of the demographics must also be recognized. This population is significantly skewed toward neonates prompting challenges with generalization given changes in hemostasis as children age. Unknown race and ethnicity were reported for a substantial part of this population, limiting utility for targeting demographics at higher risk. Lastly, several markers for thrombotic complications previously identified were missing from this study: oxygenator pressure differential, d-dimer, and thromboelastographic parameters.^8^

## Conclusion

Patients undergoing ECMO therapy who experience multiple thrombotic events during ECMO therapy face a significantly increased mortality risks, linked to clot severity. Current lab tests show limited sensitivity to predict these events the day prior in a vulnerable patient group, leading to potential ECMO circuit failures. To enhance our understanding and management of thrombotic and bleeding complications, incorporating clot severity scores into reports is essential. Additionally, contextually documenting interventions like blood product transfusions would further improve outcome analysis. These improvements could strengthen a large observational study aimed at refining anticoagulation strategies, ultimately enhancing patient outcomes in ECMO therapy.

## Supplemental Material


Supplemental Material - Comprehensive secondary analysis of thrombotic events in pediatric patients receiving extracorporeal membrane oxygenation: A prospective cohort study
Supplemental Material for Comprehensive secondary analysis of thrombotic events in pediatric patients receiving extracorporeal membrane oxygenation: A prospective cohort study by Tyler B Hughes, Rebecca N Treffalls, Wouter Koek, Heidi Dalton, Oliver Karam and Andrew D Meyer in Journal of Perfusion.
