# Supplementary material for: Comprehensive secondary analysis of thrombotic events in pediatric patients receiving extracorporeal membrane oxygenation: A prospective cohort study
Source: Perfusion. 2024 Oct 1;40(5):1202–9. doi: 10.1177/02676591241289358 (PMC12202824; doi:10.1177/02676591241289358)
Supplement: Supplemental Material - Comprehensive secondary analysis of thrombotic events in pediatric patients receiving extracorporeal membrane oxygenation: A prospective cohort study [file sj-pdf-1-prf-10.1177_02676591241289358.pdf]

Supplemental Figures and Tables

**Table S1. Clot Severity Score**	
0	None
1 (mild)	Other, hemofilter, tubing
2 (moderate)	Oxygenator change, cannula change, pump-head change, circuit change
3 (severe)	Patient thrombosis

Table S2. Demographics of Pediatric Patients on ECMO Therapy

	Thrombotic Event		
**Variable**	**Yes (N=155)**	**No (N=230)**	**P value**
**Age, n (%)**			0.178
Neonate	89 (58.0)	124 (54.0)	
Premature Neonate	21 (14.0)	16 (7.0)	
Non-Premature Neonate	68 (43.9)	108 (47.0)	
Infant	33 (21.3)	43 (18.7)	
Child	22 (14.2)	38 (16.5)	
Adolescent	11 (7.1)	25 (10.9)	
**Sex, n (%)**			0.599
Female	67 (43.0)	93 (40.0)	
Male	88 (57.0)	137 (60.0)	
**Weight, Median (IQR)**			0.121
Kg	3.50 (2.98-7.30)	3.90 (3.05-10.6)	
**Race, n (%)**			0.225
White	67 (43.2)	111 (48.3)	
African American	29 (18.7)	43 (18.7)	
Asian	9 (5.80)	8 (3.50)	
American Indian or Alaskan Native	0 (0.00)	5 (2.20)	
Unknown or Not Reported	50 (32.3)	63 (27.4)	
**Ethnicity, n (%)**			0.130
Hispanic	23 (14.8)	43 (18.7)	
Non-Hispanic	97 (62.6)	153 (66.5)	
Unknown or Not Reported	35 (22.6)	34 (14.8)	

**Primary Diagnosis, n (%)**			0.731
**Respiratory Distress or Failure**	79 (51)	111 (49)	
**Congenital Cardiovascular Disease**	51 (34)	77 (33)	
**Other Primary Diagnoses, n**			
Airway or Tracheal Abnormality	1	2	
Cancer	1	2	
Cardiac Arrest	5	7	
Arrhythmia	1	1	
Acquired Cardiovascular Disease	4	14	
Hypoxic or Anoxic Injury	0	1	
Transplant	1	5	
Pneumonia/Bronchiolitis/Pertussis	2	0	
Sepsis, SIRS, Septic Shock	8	4	
Shock	1	2	
Other	1	4	
**Cannula Size**			
**Venovenous**			0.800
Dual Lumen, n	19	43	
Fr, Median (IQR)	15 (13–19)	16 (13-23)	
Single Lumen Drain, n	1	6	
Fr, Median (IQR)	15	16 (12-23)	
Single Lumen Return, n	0	4	
Fr, Median (IQR)		15 (12-22)	
Other Unknown, n	1	4	
**Venoarterial, n**			0.667
Single Lumen Drain, n	120	171	
Fr, Median (IQR)	12 (12-16)	14 (10-16)	

Single Lumen Return, n	125	172
Fr, Median (IQR)	10 (8-12)	10 (8-12)
Unknown, n	15	9

Table S3. Demographics of Documented Circuit Clotting Events

**Clot Type, n (%)**	**N = 133**	**Averaged ECMO Day of Intervention**
Arterial Cannula Change	1 (0.60)	4
Bladder Change	9 (5.80)	5
Circuit Change	50 (32.3)	8
Circuit Tubing Change	6 (3.90)	5
CVVH Change	1 (0.60)	6
Hemofilter Change	4 (2.60)	11
Other Component Change	36 (23.2)	5
Oxygenator Change	24 (15.5)	4
Pump Head Change	1 (0.60)	6
Venous Cannula Change	1 (0.60)	9

Table S4. Demographics of Documented Patient Clotting Events

**Clot Type, n (%)**	**N = 22**	**Averaged ECMO Day of Intervention**
Intracardiac Clot	6 (3.90)	3
Intracranial Infarct	7 (4.50)	5
Limb Ischemia	9 (5.80)	4

Table S5. Univariate Analysis of Daily Laboratory Values for Single Thrombotic Event

	Thrombotic Event		
**Variable**	**Yes (N=155)**	**No (N=230)**	**P value**
**Laboratory Values, Median (IQR)**			
International Normalized Ratio	1.26 (1.14-1.41)	1.29 (1.14-1.49)	0.425
**aPTT**	**88.9 (70.7-105)**	**85.7 (68.4-103)**	**0.325**
anti-Xa (units/mL)	0.38 (0.50-0.25)	0.28 (0.19-0.42)	0.003
White Blood Cells (K/mcL)	8.89 (7.16-12.0)	8.91 (6.96-11.5)	0.654
Platelets Count (K/mcL)	104 (91.3-115)	100 (86.5-117)	0.162
Fibrinogen (mg/dL)	240 (181-317)	236 (183-305)	0.846
ATIII (%)	65.0 (49.6-83.5)	59.0 (47.9-75.9)	0.031
Plasma Free Hgb (mg/dL)	51.6 (30.0-80.4)	43.3 (16.7-68.8)	0.026
**Transfusion per Kg, Median (IQR)**			
Platelets (mL/Kg)	16.9 (11.0-26.8)	11.6 (6.27-18.3)	< 0.001
Red Blood Cells (mL/Kg)	17.6 (11.3-28.3)	13.6 (8.37-25.2)	0.001
Plasma (mL/Kg)	7.38 (3.24-13.9)	4.51 (1.09-9.87)	< 0.001
Cryoprecipitate (mL/Kg)	4.00 (0.00-8.18)	0.00 (0.00-3.71)	<0.001
Daily Heparin Dose (units/Kg)	654 (510-882)	571 (425-727)	<0.001
Hours Heparin Held (hrs)	0.00 (0.00-0.00)	0.00 (0.00-0.17)	0.455
ATIII Dose (IU/Kg)	0.00 (0.00-47.06)	0.00 (0.00-75.57)	<0.001

ECMO, Extracorporeal membrane oxygenation; E-CPR, Extracorporeal cardiopulmonary resuscitation; NICU, Neonatal intensive care unit; PICU, Pediatric intensive care unit; CICU, Cardiac intensive care unit; IQR, Interquartile Range; mL, milliliters; mcL, microliters; Kg, kilograms

Table S6. Univariate analysis of Daily Laboratory Values for Multiple Thrombotic Events

	Multiple Thrombotic Event		
**Variable**	**Yes (N=78)**	**No (N=230)**	**P value**
**Laboratory Values, Median (IQR)**			
International Normalized Ratio	1.24 (1.13-1.36)	1.29 (1.14-1.49)	0.156
anti-Xa (units/mL)	0.41 (0.25-0.55)	0.28 (0.19-0.42)	0.002
White Blood Cells (K/mcL)	8.89 (6.76-12.0)	8.91 (6.96-11.5)	0.887
Platelets Count (K/mcL)	105 (92.7-115)	100 (86.5-117)	0.097
Fibrinogen (mg/dL)	247 (192-321)	236 (183-305)	0.454
ATIII (%)	71.7 (49.7-90.1)	59.0 (47.9-75.9)	0.011
Plasma-free Hgb (mg/dL)	55.7 (33.7-80.8)	43.3 (16.7-68.8)	0.011
**Transfusion per Kg, Median (IQR)**			
Platelets (mL/Kg)	19.5 (12.5-31.5)	11.6 (6.27-18.3)	< 0.001
Red Blood Cells (mL/Kg)	19.6 (12.7-39.2)	13.6 (8.37-25.2)	<0.001
Plasma (mL/Kg)	9.42 (5.02-16.1)	4.51 (1.09-9.87)	< 0.001
Cryoprecipitate (mL/Kg)	4.89 (1.99-8.63)	0.00 (0.00-3.71)	<0.001
Daily Heparin Dose (units/Kg)	677 (551-924)	571 (425-727)	<0.001

ECMO, Extracorporeal membrane oxygenation; E-CPR, Extracorporeal cardiopulmonary resuscitation; NICU, Neonatal intensive care unit; PICU, Pediatric intensive care unit; CICU, Cardiac intensive care unit; IQR, Interquartile Range; mL, milliliters; mcL, microliters; Kg, kilograms

Table S7. Scoring and Hospital Mortality

	Thrombotic Event	No Thrombotic Event	
n	155	230	
Clot Severity Score (Median, IQR)	3 (2-4)	0	
**Hospital Mortality**	**Alive**	**Deceased**	**Total**
Score ≥ 4	22	33	55
Score < 4	202	128	330
	**OR**	**CI 95%**	
Score ≥ 4	2.37	1.32-4.24	

IQR, interquartile range; CI 95%, confidence interval 95%
